# Comparative analysis of PFASs concentrations in fur, muscle, and liver of wild roe deer as biomonitoring matrices

**DOI:** 10.3389/fvets.2024.1500651

**Published:** 2024-12-23

**Authors:** Susanna Draghi, Giulio Curone, Roberta Risoluti, Stefano Materazzi, Giuseppina Gullifa, Angela Amoresano, Michele Spinelli, Carolina Fontanarosa, Radmila Pavlovic, Alberto Pellegrini, Marco Fidani, Petra Cagnardi, Federica Di Cesare, Francesco Arioli

**Affiliations:** ^1^Department of Veterinary Medicine and Animal Sciences, University of Milan, Lodi, Italy; ^2^Department of Chemistry, Sapienza University of Rome, Rome, Italy; ^3^Department of Chemical Sciences, University of Naples Federico II, Napoli, Italy; ^4^I.N.B.B., Istituto Nazionale Biostrutture e Biosistemi, Rome, Italy; ^5^Proteomics and Metabolomics Unit, San Raffaele Scientific Institute, Milan, Italy; ^6^UNIRELab, Settimo Milanese, Italy

**Keywords:** biomonitoring, perfluoroalkyl substances, high-resolution mass spectrometry, ecotoxicology, wildlife, endocrine disruptors, roe deer, environmental pollution

## Abstract

**Background:**

Recently, environmental pollution has become a significant concern for human, animal, and environmental health, fitting within the “One Health” framework. Among the various environmental contaminants, per- and polyfluoroalkyl substances (PFASs) have gathered substantial attention due to their persistence, bioaccumulation, and adverse health effects. This study aimed to compare the levels of 12 PFASs in the fur, liver, and muscle of wild roe deer to evaluate the feasibility of using fur as a non-invasive biomonitoring matrix.

**Methods:**

A total of 20 male and 20 female roe deer aged between 12 and 24 months were randomly sampled from a hunting area in Northern Italy. Samples of fur, muscle, and liver were collected post-mortem, and PFAS concentrations were measured using a validated UHPLC-HRMS method.

**Results and discussion:**

The results indicated significant differences in PFAS concentrations among the three matrices. Fur, although easier to sample and store, showed highly variable PFAS levels, with different detection frequencies compared to the muscle and liver. PFASs such as PFHxA were more frequently detected in fur than in the liver and muscle, while compounds such as PFBA, PFPeA, PFHpA, PFDA, PFHxS, 6-2 FTS, and 8-2 FTS were less frequently detected in fur. In conclusion, while fur presents many practical advantages for biomonitoring, such as non-invasive sampling and stability, its use is complicated by varying detection frequencies and concentration levels. These aspects, together with the use of a single sampling technique, can be considered a limitation of the study. Notably, compounds such as PFOA, PFNA, and PFOS showed partially similar detection frequencies across the matrices, suggesting potential interest for further research. This study offers new perspectives on the use of fur for environmental monitoring, highlighting the need for more extensive research to understand the relationship between PFAS concentrations in fur and other biological matrices. Future studies should focus on methodological improvements in extraction and quantification techniques for PFASs in fur to enhance their reliability as a biomonitoring tool.

## 1 Introduction

Recently, environmental pollution has become a concern for human, animal, and environmental health within the context now defined as “One Health” (1). Environmental contaminants are defined as substances introduced into the environment due to human activity or natural processes that can harm ecosystems, organisms, or human health. These include pollutants such as metals, pesticides, polychlorinated biphenyls (PCBs), dioxins, plastics, per- and polyfluoroalkyl substances (PFASs), and hydrocarbons (2). They often originate from industrial processes, agricultural runoff, waste disposal, or atmospheric deposition and can accumulate in water, soil, air, and biological systems (3). Once released into the environment and comes into contact with living organisms, if the rate of absorption exceeds the rate of elimination, bioaccumulation occurs (4). Another characteristic of environmental pollutants is their entry into the food chain, leading to the phenomenon known as biomagnification, which describes the increasing concentration of these substances as they move up the food chain. Predators at higher trophic levels accumulate higher concentrations of contaminants because they consume multiple prey, each containing accumulated pollutants (4). Environmental contaminants that can bioaccumulate and biomagnify, and thus have a long half-life, are known as persistent organic pollutants (POPs). These substances remain stable in the environment, with extended half-lives in soil, sediments, air, or living organisms (5). Although there is no universally agreed-upon threshold for the half-life duration required for a pollutant to be deemed *persistent*, in practical terms, a POP may have a half-life ranging from years to decades in soil or sediments and lasting several days in the atmosphere (6). The bioaccumulation and biomagnification of environmental pollutants in living organisms often result in adverse health effects (7).

In general, exposure to xenobiotics is influenced by numerous factors related to both the living organism and the compound; hence, environmental monitoring may not be sufficient to determine the real exposure at the biota level, as stated by Rendon Lugo et al. and Zhou et al. (8, 9). Indeed, standard environmental monitoring can detect the presence of contaminants, but does not provide information on the biological effects these contaminants have on living organisms. Wild mammals can show signs of physiological stress, behavioral changes, reduced fertility, and other biological responses that may indicate the real impact of the contaminants (10). Moreover, since wild mammals often share the same ecosystems and food resources with humans, they can serve as indicators for understanding the potential health effects on humans (10). Finally, another particularly useful aspect is the possibility of obtaining a more comprehensive picture of the distribution and concentration of contaminants in the environment (11, 12). Wild mammals, through their movements and behaviors, can accumulate contaminants from various sources and geographical areas (13). Over the past few decades, numerous wild mammals have been identified as potential sentinels for monitoring the presence of xenobiotics and have been used to conduct biomonitoring studies. Some examples include herbivorous and omnivorous macromammals such as the wild boar (14), otter (15), deer (16), and roe deer (17) and herbivorous and omnivorous micromammals such as shrews (18), hedgehogs (19), and bats (20). The European roe deer, among wild mammals, has been recognized as a suitable bioindicator due to its unique behavioral traits. These include small home ranges (16–80 ha) and high behavioral adaptability, enabling it to thrive in diverse habitats, including those heavily influenced by human activities (21). Roe deer primarily consume leaves, young shoots, berries, and grass, favoring easily digestible forage. Studies have shown that pollutant levels in their muscles correlate with their dietary intake (22, 23). In the past, this animal has already been used as a bioindicator for pesticides (24), fluoride, lead (25), and toxic trace elements (26).

Among environmental contaminants, PFASs are xenobiotics receiving considerable attention recently from the scientific and public communities and also frequently considered in biomonitoring programs. Per- and polyfluoroalkyl substances are emerging POPs of anthropogenic origin (27). They contain between 5,000 and 10,000 compounds (28), regularly used in various industrial processes such as plasticizers, polymerizing acids, and flame retardants (29). Their worldwide use is attributed to their chemical–physical characteristics, ensuring high thermal, chemical, and biological stability (30, 31). These same characteristics prevent their degradation when dispersed into the environment, leading to their entry into the food chain and subsequent bioaccumulation in biota (32). Over the decades, their bioaccumulation in living organisms has been found to have numerous serious adverse health effects, both in humans and animals. A growing number of studies on humans and animals, and mammals in general, indicate that exposure to PFASs leads to the disruption of endocrine functions and metabolism (33), impairment of liver and thyroid hormones (34), impact on renal physiology (35) and bones (36), together with their immunotoxicity with immunosuppressive effects (37, 38).

Till date, the identification and quantification of PFASs, both in humans and animals, have been mainly carried out in tissues and body fluids such as muscle, liver (39) blood, urine, and feces (40). All these matrices allow for precise quantification but have ethical and practical implications. They are difficult to obtain as sampling requires invasive procedures with physical or chemical restraint of animals or must occur post-mortem, during necropsy, or at the slaughterhouse (20, 41). The biological matrices traditionally used also present practical problems of transportation and preservation since they degrade very easily (41). In recent decades, to overcome many of these inconveniences, many new matrices collected non-invasively have been used to identify the presence of PFASs in living organisms. Non-invasive sampling, as the name suggests, involves the collection of tissues without sacrificing the animal and with minimal pain or stress induction (20, 42). The matrices mainly used have been hair in humans and fur in animals; however, other matrices have also been identified in animals, for example, feathers in birds (43). These matrices are much simpler to sample as they do not require any invasive procedure (44). Fur, if stored away from light, humidity, and heat sources, is a stable matrix (12). Both fur and hair have been frequently used for identifying exposure to xenobiotics despite the lack of pharmacokinetic or toxicokinetic studies (45). Indeed, substances circulating in the blood are incorporated into the fur through the follicle and distributed along its length as it grows. Finally, but fundamentally, fur allows for the identification of exposure to a xenobiotic over a broader time window, unlike the immediate timeframe of other regularly used matrices (44).

Hair and fur have been used to identify exposure to various xenobiotics and drugs, but to the best of our knowledge, only one study has quantified the presence of PFASs in the fur of dogs (46). For this reason, the objective of this study was to quantify 12 PFASs in the fur, liver, and muscle of wild roe deer to understand whether the fur reflects the PFAS content in the organism and, therefore, whether the fur of wild animals can be used as a biomonitoring tool for the environmental presence of PFASs.

## 2 Materials and methods

### 2.1 Animals and sample collection

This study included 40 roe deer (20 males and 20 females), aged between 12 and 24 months. A specific area of Northern Italy, which presented a well-structured hunting activity, was chosen, and the sampling procedures were conducted during the hunting season from June to August. The hunting area is characterized by an altitude ranging from 500 to 900 m above sea level. There are cultivated areas, sporadically inhabited centers, wooded areas with oaks and beeches, and pastures in the higher altitude zones. The animal culling area was georeferenced. The authors, before starting the procedure, requested and obtained the study approval as a non-experimental project by the Institutional Animal Care and Use Committee of Università degli Studi di Milano (Permission OPBA_26_2022). Tissues from the 40 roe deer were collected at the hunting meat processing plants after the culling during the regular hunting activities. From each animal, before starting the slaughtering procedures, an area of 10 cm^2^ was shaved close to the skin with an electric shaver, behind the costal arch, on the left side of the animal. Fur was preserved and wrapped in aluminum foil in a dry and shaded place until the analysis. During the slaughtering procedures, 100 g of muscle, from the *longissimus lumborum et thoracis* on the left side of the carcass, and 100 g of liver were collected. The tissue samples were stored in 50-mL glass conical centrifuge tubes (Corning Incorporated, Corning, NY, USA) at −20°C.

### 2.2 Chemicals and reagents

Acetic acid, acetone, sodium dodecyl sulfate (SDS), and methanol of analytical grade were purchased from Merck (Merck KGaA, Darmstadt, Germany). The internal standards (IS), C-labeled, MPFOS (sodium perfluoro-1-[1,2,3,4-13C4]octanesulfonate) and MPFNA (perfluoro-n-[1,2,3,4,5-13C5]nonanoic acid) and the ISOmix (ISO21675:2019 native stock solution) were purchased from Wellington Laboratories INC (345 Southgate Drive, Guelph, Ontario N1G3M5, Canada). Individual stock standard solutions of IS were prepared at a concentration of 1 ppm in MeOH and stored at −20°C. Working solutions were prepared daily by diluting the stock standard solutions in methanol.

### 2.3 Extraction procedure and UPLC-HRMS

The protocol used for the sample preparation for the analysis of fur was previously described by Makowska et al. and Martin et al. (46, 47), with a few modifications. An aliquot of 0.2 g of the sample was weighed and placed into a glass tube with a conical bottom to perform a fourfold washing to remove exogenous substances: first, 10 ml of ultrapure water was added, gently vortexed, and sonicated for 5 min. Then, 10 ml of SDS 0.1% was added. The samples were gently vortexed and sonicated for 5 min. In the end, two subsequent washing steps were carried out with 10 ml of ultrapure water, each one followed by a 5-min sonication. After the washing, the fur was dried at room temperature on blotting paper. After complete drying, 0.1 g of fur from each sample was weighed, cut into small fragments approximately 2–3 mm in length, and placed into a new glass tube. To the aliquots of fur, 1 ng of IS and 2 ml of a mixture of methanol and acetic acid (MeOH/HAc 85:15 v/v) were added, gently vortexed, and incubated at 38°C for 12 h. The samples were cooled at room temperature and then 3 mL of acetone was added. After a 15-min sonication step, the samples were centrifuged for 10 min at 2,900 × *g*. fThe supernatant was transferred into a clean glass tube using a glass Pasteur pipette and evaporated until dry under vacuum in a centrifugal evaporator at a temperature of 55°C. In the end, the extract was reconstituted by adding 100 μl of MeOH and 100 μl of the mobile phase (90% water with 20 mM ammonium formate and 10% MeOH), vortexed, and transferred into vials for UPLC-HRMS. For the extraction of PFASs from muscle and liver samples, a method already validated by the authors in other biological matrices was used (39, 48, 49). The instrumental method, based on the recent study by Draghi et al. (49), used a Vanquish system equipped with a binary pump, auto-sampler, and a thermostatted compartment for two columns (Thermo Fisher Scientific, Waltham, MA, USA), coupled to a Thermo Q Exactive Orbitrap™ (Thermo Fisher Scientific, Waltham, MA, USA) with a heated electrospray ionization source. Instrumental parameters for UPLC-HRMS are detailed in the Supplementary material. Post-run chromatograms and spectra were processed using Xcalibur™ 4.3 software (Thermo Fisher Scientific, Waltham, MA, USA) for data interpretation.

### 2.4 Method validation

Validation was conducted for perfluorobutanoic acid (PFBA), perfluoropentanoic acid (PFPeA), perfluorohexanoic acid (PFHxA), perfluoroheptanoic acid (PFHpA), perfluorooctanoic acid (PFOA), perfluorononanoic acid (PFNA), perfluorodecanoic acid (PFDA), perfluorobutanesulfonic acid (PFBS), perfluorohexanesulfonic acid (PFHxS), perfluorooctanesulfonic acid (PFOS), 6:2-fluorotelomersulfonic acid (6-2 FTS), and 8:2-fluorotelomersulfonic acid (8-2 FTS), in accordance with the updated SANTE guidelines 11,312/2021 (75), by assessing selectivity, limit of detection (LOD), limit of quantification (LOQ), and matrix effect. The method’s selectivity was confirmed by evaluating interference peaks in blank samples near the expected PFAS retention times. To prevent analytical misinterpretation due to potential PFAS contamination in extraction and purification materials, eight procedural blanks without matrix were prepared for each extraction session. Quality assurance/control (QA/QC) was carried out by analyzing five matrix blank samples to determine PFAS contributions in unfortified matrices, and adjusting final concentrations if necessary. Matrix-matched calibration curves (10–100 pg g^−1^) were constructed by spiking blank samples with standard mixtures. LOD and LOQ were calculated using the equations LOD = 3.3 SD/b and LOQ = 10 SD/b, where SD is the standard deviation of the intercept for low concentration levels and b is the slope of the regression line from the principal calibration curve. The matrix effect was quantified by comparing peak areas of PFASs spiked after blank sample extraction to those of standards in solution, expressed as a percentage. The validation procedures for the liver and muscle were carried out in a previous work by this research group (39). The validation parameters for PFASs, considered in this study, in the liver and muscle are shown in Supplementary Table S1. The validation parameters for fur are shown in Table 1.

**Table 1:** Validation parameters for PFASs detection in fur.

Compound	Formula	Parent exact mass m/z	RT (min)	LOD (pg/g)	LOQ (pg/g)	Linearity	R^2^	Recovery (%)	Matrix effect (%)	CV intraday (%)	CV Interday (%)
PFBA	C4HF7O2	213	3.56	0.167	0.506	y = 0.0233x-0.001	0.999	106	85	10	16
PFPeA	C5HF9O	263	9.28	1.573	4.767	y = 0.0136x-0.0008	0.983	98	90	12	15
PFHxA	C6HF11O2	313	13.03	1.999	6.059	y = 0.0181x-0.0025	0.978	100	102	9	17
PFHpA	C7HF13O2	363	15.19	0.679	2.058	y = 0.0331x-0.0022	0.976	95	97	14	20
PFOA	C8HF15O2	413	16.72	0.911	2.760	y = 0.0452–0.0035	0.992	108	102	11	16
PFNA	C9HF17O2	463	17.93	0.846	2.565	y = 0.0522x-0.0017	0.991	99	98	10	18
PFDA	C10HF19O2	513	18.12	0.128	0.387	y = 0.1619x-0.0009	0.999	101	116	10	19
PFBS	C4HF9O3S	299	10.61	1.557	4.717	y = 0.0393x-0.0004	0.998	97	114	13	20
PFHxS	C6HF13O3S	399	15.37	0.826	2.503	y = 0.0463x-0.0015	0.998	95	83	10	19
PFOS	C8HF17O3S	499	17.91	0.755	2.289	y = 0.0546x + 0.0001	0.992	102	100	11	16
6-2FTS	C8H5F13O3S	427	20.29	0.164	0.496	y = 0.1038x + 0.0716	0.998	106	95	9	15
8-2FTS	C10H4F17O3S	527	19.59	31.343	94.978	y = 0.05104x-0.0004	0.996	98	106	12	20

### 2.5 Statistical analysis

At first, a preliminary statistical evaluation was performed using the Shapiro–Wilk test, which revealed that data were not normally distributed; thus, a non-parametric statistical evaluation was applied. In particular, the Kruskal–Wallis one-way (non-parametric ANOVA) analysis was used to check differences between the three datasets (fur, muscle, and liver), followed by all pairwise multiple comparison processes (Dwass–Steel–Critchlow–Fligner method). Statistical analyses were performed using GraphPad Prism 8 ® software (La Jolla, CA, USA). A *p*-value of 0.05 was set as significant, while a *p*-value of ≤0.1 was considered a tendency.

## 3 Results

**Figure 1:**
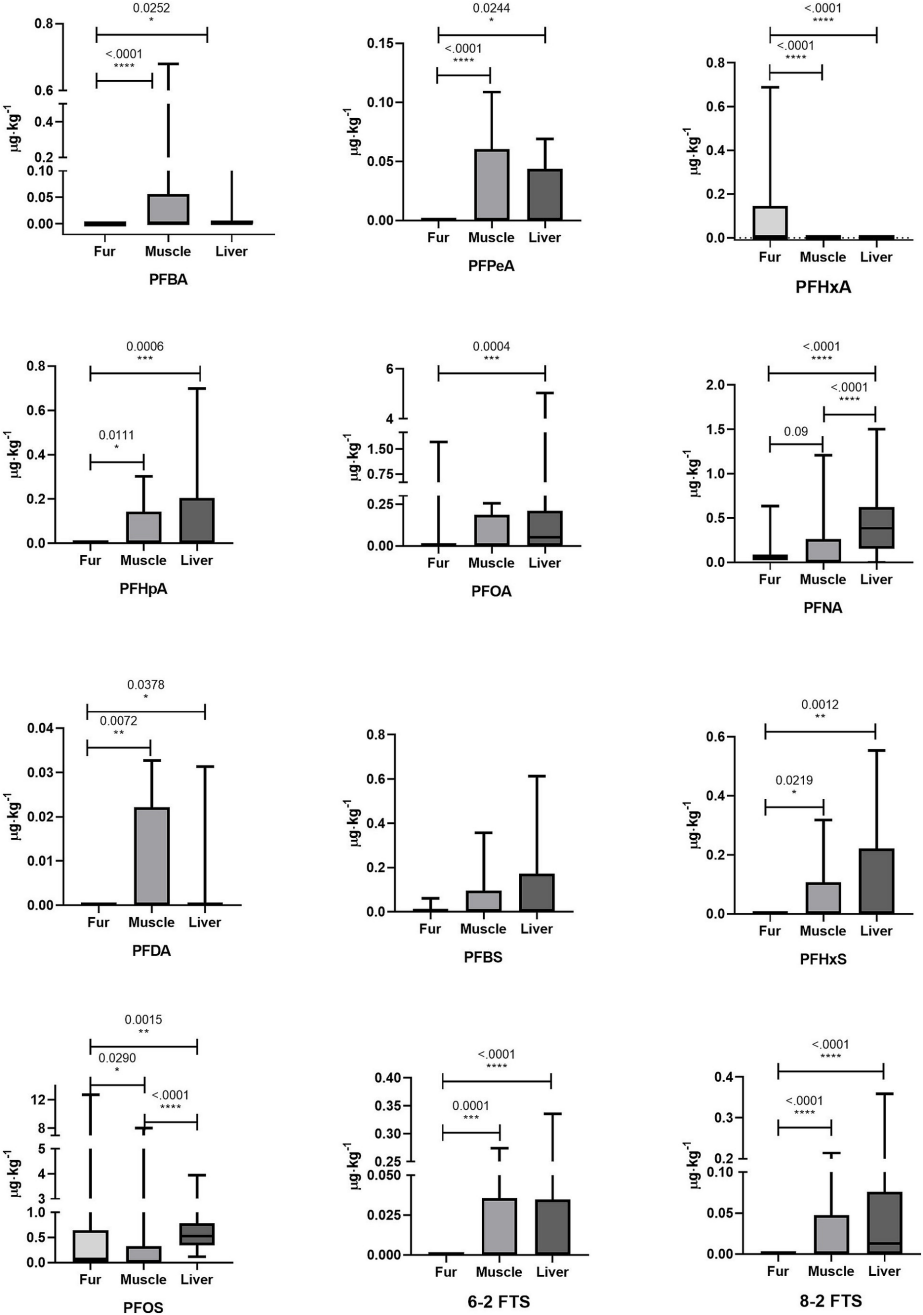
Graphical representation comparing the concentrations of PFASs in the fur, muscle, and liver of roe deer.

Mean, median, minimum, and maximum levels of PFAS concentration and statistically significant differences between matrices are reported in Table 2. The differences are reported graphically in Figure 1.

**Table 2:** PFASs content in the fur, muscle, and liver of roe deer.

	Matrix	Mean ± SD	Median	Minimum–maximum	25th perc	75th perc	DF %	*p*-value
PFBA	Fur	N.D.						<0.0001 ^a^
	Muscle	0.051 ± 0.119	0.000	0–0.68	0.000	0.050	40.0	<0.005 ^b^
	Liver	0.019 ± 0.039	0.000	0–0.170	0.000	0.002	25.0	
PFPeA	Fur	N.D.						<0.0001 ^a^
	Muscle	0.026 ± 0.032	0.000	0–0.109	0.000	0.059	42.5	<0.005 ^b^
	Liver	0.016 ± 0.028	0.000	0–0.069	0.000	0.015	25.0	
PFHxA	Fur	0.071 ± 0.134	0.000	0–0.687	0.000	0.145	32.5	
	Muscle	0 ± 0	0.000	0–0.002	0.000	0.000	2.5	
	Liver	0 ± 0	0.000	0–0	0.000	0.000	0.0	
PFHpA	Fur	N.D.						<0.05 ^a^
	Muscle	0.065 ± 0.116	0.000	0–0.303	0.000	0.047	25.0	<0.001 ^b^
	Liver	0.079 ± 0.148	0.000	0–0.698	0.000	0.197	35.0	
PFOA	Fur	0.144 ± 0.414	0.000	0–1.710	0.000	0.000	15.0	
	Muscle	0.069 ± 0.101	0.000	0–0.255	0.000	0.178	40.0	<0.001 ^b^
	Liver	0.258 ± 0.829	0.054	0–5.036	0.000	0.209	67.5	
PFNA	Fur	0.080 ± 0.103	0.042	0.032–0.632	0.033	0.079	100.0	<0.1 ^a^
	Muscle	0.112 ± 0.227	0.003	0–1.207	0.000	0.130	62.5	<0.0001 ^b^
	Liver	0.414 ± 0.322	0.384	0–1.504	0.157	0.604	92.5	<0.0001 ^c^
PFDA	Fur	N.D.						<0.05 ^a^
	Muscle	0.008 ± 0.013	0.000	0–0.033	0.000	0.007	25.0	<0.05 ^b^
	Liver	0.006 ± 0.012	0.000	0–0.031	0.000	0.000	20.0	
PFBS	Fur	0.004 ± 0.013	0.000	0–0.062	0.000	0.000	17.5	
	Muscle	0.065 ± 0.118	0.000	0–0.356	0.000	0.034	32.5	
	Liver	0.082 ± 0.152	0.000	0–0.613	0.000	0.064	30.0	
PFHxS	Fur	N.D.						<0.05 ^a^
	Muscle	0.061 ± 0.110	0.000	0–0.318	0.000	0.036	25.0	<0.001 ^b^
	Liver	0.094 ± 0.163	0.000	0–0.554	0.000	0.214	30.0	
PFOS	Fur	0.912 ± 2.258	0.067	0–12.717	0.000	0.642	70.0	
	Muscle	0.296 ± 1.263	0.000	0–8.020	0.000	0.277	47.5	<0.001 ^b^
	Liver	0.666 ± 0.636	0.526	0.114–3.941	0.351	0.782	100.0	<0.0001 ^c^
6-2 FTS	Fur	N.D.						<0.0001^a^
	Muscle	0.037 ± 0.070	0.000	0–0.274	0.000	0.026	42.5	<0.0001^b^
	Liver	0.033 ± 0.071	0.000	0–0.335	0.000	0.033	47.5	
8-2 FTS	Fur	N.D.						<0.0001^a^
	Muscle	0.043 ± 0.063	7E-05	0–0.213	0.000	0.045	50.0	<0.0001^b^
	Liver	0.049 ± 0.077	0.013	0–0.359	0.000	0.066	50.0	

Concentrations are exposed in μg.kg^−1^. DF%: detection frequencies expressed in %. a: difference between fur and muscle; b: difference between fur and liver; c: difference between muscle and liver; N.D. = not detected.

In this study, PFNA and PFOS resulted in significantly higher levels in the liver than in muscle and significantly lower levels in the fur than in the other matrices. Specifically, PFNA concentration detected in fur was 0.080 ± 0.103 μg⋅kg^−1^, in muscle was 0.112 ± 0.227 μg⋅kg^−1^, and in liver was 0.414 ± 0.322 μg⋅kg^−1^. PFOS concentration detected in fur was 0.912 ± 2.258 μg⋅kg^−1^, in muscle was 0.296 ± 1.263 μg⋅kg^−1^, and in liver was 0.666 ± 0.636 μg⋅kg^−1^. All the other compounds showed only tendencies to be higher in the liver than in the muscle. In fur, PFBA, PFPeA, PFHpA, PFDA, PFHxS, 6–2 FTS, and 8–2 FTS were not detected and thus significantly lower than in the liver and muscle. The compound PFBS was quantified in fur but did not show any statistical difference between the evaluated matrices (fur concentration: 0.004 ± 0.013 μg⋅kg^−1^; muscle concentration: 0.065 ± 0.118 μg⋅kg^−1^; liver concentration: 0.082 ± 0.152 μg⋅kg^−1^). PFHxA was detected in fur (0.071 ± 0.134 μg⋅kg^−1^) and in one sample of muscle (0.002 μg⋅kg^−1^), even though no statistically significant differences were identified in comparison with the liver and muscle. The compound with the most similar detection frequencies among the three matrices was PFOS, although it showed a significantly lower concentration in fur than in the muscle and liver.

## 4 Discussion

To the best of our knowledge, this is the first study on wildlife that compares the concentration of 12 different PFASs in different matrices (fur, liver, and muscle) from the same animal. This work interprets the One Health perspective by using the roe deer as a biomonitoring species to identify the presence of PFASs in an environment where humans and animals live in close contact, thus proposing this wild species as a sentinel of PFAS pollution.

In this study, we identified PFBA, PFPeA, PFHxA, PFHpA, PFOA, PFNA, PFDA, PFBS, PFHxS, PFOS, 6-2 FTS, and 8-2 FTS in the three analyzed matrices (liver, muscle, and fur). The concentrations of PFNA and PFOS were found to be significantly higher in the liver than in the muscle, consistent with those reported by the authors for liver and muscle roe deer samples collected in a neighboring area (39). The mean and median concentrations in the liver and muscle were also similar. Differently, in the present study, FOSA was not identified in any of the three considered matrices, while PFHxA was identified in the fur and in one muscle sample. The concentrations of PFASs in this study were also compared to those reported in other species. For example, in wild boar liver, the mean concentrations of PFOS and PFOA were 215 and 8.18 μg·kg^−1^, respectively (50). These differences could be explained by interspecies variations, particularly concerning feeding habits, digestive physiology features, and home ranges. Moreover, the concentrations of PFASs found in roe deer tissues in our study were lower than those reported by Falk et al. for roe deer in Germany (17). A study by Falk covered a larger and more diverse sampling area, encompassing forested, agricultural, and suburban habitats, and was conducted during a historical period (1989–2010) when stricter PFAS regulations were not yet in place. Conversely, our sampling focused on a more restricted area and a single hunting period. Interestingly, PFAS concentrations in roe deer muscle reported in our study were lower than those reported in other species, such as duck (51), cattle (52), and pig (53). This suggests potential interspecies differences that may stem from varying ecological behaviors, exposure routes, and physiological mechanisms of PFAS metabolism (39). In general, PFASs are primarily distributed to protein-rich tissues such as the liver, kidneys, and serum (54, 55), unlike similar hydrophobic contaminants such as PAHs and PCBs (56), which accumulate in adipose tissue. This distribution kinetics has been attributed to their high affinity for proteins, particularly plasma albumins synthesized in the liver (57).In addition, their chemical type causes the tissue-serum partition coefficient to vary from compound to compound. For example, PFOS, PFOA, and PFBS accumulate more in protein-rich tissues such as the liver, whereas PFBA and PFHxS are more distributed in the serum (57, 58). The physicochemical properties of PFASs and their affinities for the aforementioned protein matrices have made blood, serum, muscle, and liver the preferred matrices for quantifying PFASs in both humans and animals over the past decades (59). Obtaining these types of samples *in vivo* is impossible without sometimes extremely invasive interventions, which carry significant ethical implications. For this reason, alternative matrices are now being explored to identify and quantify PFASs (46).

In humans, many studies have reported the use of hair (60); conversely, to the best of our knowledge, and at the time of writing this article, a single veterinary study has been conducted by Makowska et al. (46) on domestic animals, specifically on dog fur. In the study by Makowska et al. (46), dogs were identified as sentinels for PFAS exposure of their owners as they share the same living conditions and are consequently exposed to similar environmental contaminants (61). As described by many authors, one of the peculiar characteristics of PFASs is their widespread environmental distribution, even far from their production and usage sites (62, 63). This is why wild animals are widely used as sentinels for their environmental presence (64). In this study, roe deer were evaluated as they are free-living wild mammals, which are widely distributed across various environments, including those heavily influenced by anthropogenic activities, making them easily exposed to PFASs (39). In the context of this study, the typical anthropogenic activities exploited within the evaluated geographical area that can contribute to the release of substances such as pesticides, pharmaceutical residues, and trace elements in the environment, include agriculture and livestock farming.

Regarding the identified concentration ranges, when compared with the study by Makowska et al. (46) in dogs, our results indicate lower concentrations in roe deer fur. Moreover, Makowska et al. (45) identified PFBuA, PFPeA, PFHxA, PFHpA, PFOA, and PFOS; in this study, we also identified PFNA, PFDA, and PFBS, obtaining a more comprehensive panel of compounds, including shorter-chain perfluoroalkyl substances and sulfonated compounds. Certainly, comparing different species is complex because the existing toxicokinetic data on PFASs show significant interspecies differences in some toxicokinetic parameters among different compounds (65). The first difference in absorption concerns the digestive system, a physiological feature able to affect the bioavailability of xenobiotics. The roe deer is a ruminant, a selective browser of concentrated forage (66, 67), whereas the dog has a monogastric digestive system and can currently be considered omnivorous (67). The roe deer, being herbivorous, is at the second level of the food chain, whereas the dog is at the third level of the food chain (64). This implies differences in the biomagnification of PFASs. The differences in feeding behavior, food chain position, and digestive physiology can be considered primary factors influencing the absorption of PFASs (58). Another factor to consider is the living environment. The dogs sampled in the study by Makowska et al. (46) are from urban areas, whereas the roe deer analyzed in this study are free-living animals from sparsely populated rural areas, likely resulting in lower exposure to PFASs. Finally, the average lifespan; the dogs analyzed were aged between 3 and over 10 years, while the roe deer used in this study (to minimize variability and focus on matrix comparison) were aged between 12 and 24 months. As reported in other studies, age is an influencing factor of exposure; older animals are exposed to xenobiotics for a longer time and consequently present higher concentrations of contaminants in their tissues (21, 67). All these factors could explain the wide difference observed.

In many studies, fur has been considered a highly accessible matrix for monitoring wild animals, both in terms of environmental toxicology and for different studies related to the general well-being of free-living animals (68). For wildlife, other biological matrices such as blood and muscle are difficult to sample in vivo, whereas in many studies, fur has been considered for non-invasive biomonitoring. An example is the method proposed by Henry et al. (68) for elusive small mammals, which involves placing strips of packing tape arranged in a web-like fashion along travel routes in the pikas’ habitat (68).

For other compounds, such as trace elements, hair has been identified as an excellent matrix for their quantification (69, 70). The availability of studies related to PFASs is very scarce and mainly related to studies conducted on human hair (61). The main problem with using hair as a monitoring tool for PFAS exposure is understanding whether it accurately reflects concentrations in the more commonly used matrices. Alves et al. (60) reported a high variability in PFAS concentrations in hair (ranging from <LOQ to 46 ng/g). As shown in Table 2, also in our case, the concentrations and standard deviations of the compounds quantified in hair are very broad. A study by Li et al. (71) showed that many PFASs identified in hair have a lower concentration and a much lower detection frequency (DF%) than those in urine or nails (60, 71). Our data are also consistent with this, as shown in Table 2 for PFBA, PFPeA, PFHpA, PFDA, PFHxS, 6-2FTS, and 8-2FTS. However, surprisingly, PFHxA was identified much more frequently in hair compared to liver and muscle. Only PFOA, PFNA, and PFOS have partially similar detection frequencies (DF%) in the three analyzed matrices, again in agreement with other studies (60, 71).

These differences are likely due to the so-called matrix effect and the type of hair, which can significantly influence quantification (60). It is widely recognized that the structure of hair (and fur) is a crucial parameter for the incorporation of both drugs and environmental contaminants (72). Despite this, as previously mentioned, PFASs bind to serum proteins and are not lipophilic like other POPs (57), making their quantification in hair reasonable and straightforward (73, 74). Another factor to consider is that hair is a complex matrix with multiple compartments, such as the skin above the hair bulb from which pollutants can access, diffusion from blood to the actively growing follicle, from secretions of the apocrine and sebaceous glands (sweat and sebum), or from the external environment during the hair growth cycle (74). Another factor that could be considered involves the deposition of PFASs on the outer surface of the hair/fur. However, in the case of this study, it can be excluded because the procedure involves multiple washes coupled to sonication before the extraction, as described in the Materials and Methods section. In fact, the wash protocol is always necessary to eliminate the contamination of the fur’s outer face and to guarantee the detected compounds derived from the inside of hair/fur. Conversely, a factor that could have influenced the concentration of PFASs is the absence of the hair bulb. Indeed, our hair samples were shaved close to the skin and not plucked. The hair bulb is directly supplied by the bloodstream and could therefore present a higher concentration (45). Sampling hair with the bulb requires a slightly more invasive sampling procedure that would cause pain and stress to the sampled subject, thus making this type of sampling more complex in the case of wild animals and those raised in captivity. In addition, the collection of fur samples from wild animals in their natural habitat without direct contact, with tools such as hair traps, would result in any case in the sampling of hair without the bulb. For these reasons, the proposed method and the considerations raised in this study can be considered applicable to other non-invasive sampling situations of fur matrix.

Finally, neither in this study nor, to the best of our knowledge, in other studies, have the differences in accumulation in hair based on the carbon chain length of PFASs been considered. This type of study has been done on other matrices such as milk (49) and blood (61, 65). The factors described above may explain the variability in identified concentrations and the difference between the liver and muscle, which have a single route of exposure, namely blood perfusion.

## 5 Conclusion

This study described a successful method validation for identifying 12 PFASs in roe deer fur, and detection frequencies and concentrations in fur for most substances were generally lower than those found in liver and muscle. Nevertheless, this biological matrix demonstrates many positive aspects for biomonitoring purposes, such as ease of sampling and simplicity of storage, but the determination of how and to what extent PFASs can distribute to fur is yet unknown. Given the results obtained for PFOA, PFNA, and PFOS, this study offers new perspectives, requiring a greater amount of research of this type to understand the relationship between concentrations in fur compared to other matrices such as blood, urine, and feces, and studies aimed at methodological improvement of extraction and quantification techniques for PFASs in this promising matrix, which might include a larger sample aliquot of fur for analysis or the use of different solvents for the washing steps. Finally, further investigation will be necessary to compare the outcomes of different fur sampling techniques (e.g., shaving and plucking with the bulb) and to explore the spatial–temporal trends of PFAS presence in this matrix.

## Data Availability

The original contributions presented in the study are included in the article/Supplementary material, further inquiries can be directed to the corresponding author.
