# Supplementary material for: Comparative analysis of PFASs concentrations in fur, muscle, and liver of wild roe deer as biomonitoring matrices
Source: Front Vet Sci. 2024 Dec 23;11:1500651. doi: 10.3389/fvets.2024.1500651 (PMC11701231; doi:10.3389/fvets.2024.1500651)
Supplement: Supplementary file 1 [file Table_1.docx]

Supplementary Material

# Instrumental parameters used for UPLC-HRMS

All PFASs were chromatographically separated by using a Raptor ARC-18 5 um EXP guard column (Restek, Bellefonte, PA, USA). Moreover, a CMB WR C18 50 × 4.6 mm, 10 µm (PerkinElmer Italia SPA, Milan, Italy), was introduced before the injector to allow delaying of eventual PFASs already present in the system. The mobile phase consisted of phase A (20 mM aqueous ammonium formate) and B (MeOH). The gradient started with 20% B, which reached 95% B at the 20th min and was kept in this condition for 10 min. At the 30th minute, the initial conditions (20% B) were reached and kept for 4 min for riequilibration. The run was performed at 0.3 mL min-1, with a total duration of 35 min. Regarding the detector parameters, the capillary and vaporizer temperatures were set at 330 and 280 °C, respectively, the sheath and auxiliary gas were set at 5 and 15 arbitrary units, and the electrospray voltage was set at 3.50 kV, operating in negative mode. The full scan (FS) acquisition (70,000 FWHM resolution, scan range 200–950 m/z, 1E6 of automatic gain control AGC, maximum injection time of 200 ms) was combined with a data-independent acquisition (DIA) mode for the confirmatory response, based on an inclusion list that operated at 35,000 FWHM resolution, 5E4 AGC target, maximum injection time of 100 ms, and isolation window of 2 m/z.

# Table S1. Validation parameters for PFASs detection in muscle and liver.

| **Compound** | **Formula** | **Parent Exact Mass m/z** | **RT (min)** | **LOD (pg/g)** | **LOQ (pg/g)** | **Linearity** | **R^2^** | **Recovery (%)** | **Matrix Effect (%)** | **CV Intraday (%)** | **CV Interday (%)** |
| --- | --- | --- | --- | --- | --- | --- | --- | --- | --- | --- | --- |
| PFBA | C4HF7O2 | 213 | 3.71 | 0.615116 | 1.863989 | y = 0.0706x + 0.0067 | 0.978 | 108 | 83 | 11 | 19 |
| PFPeA | C5HF9O | 263 | 9.64 | 0.402452 | 1.219553 | y = 1.1245x - 0.0501 | 0.979 | 101 | 82 | 12 | 15 |
| PFHxA | C6HF11O2 | 313 | 13.37 | 2.715292 | 8.228159 | y = 0.1497x + 0.0012 | 0.999 | 98 | 100 | 10 | 18 |
| PFHpA | C7HF13O2 | 363 | 15.53 | 0.247166 | 0.748987 | y = 0.1842x + 0.0014 | 0.999 | 89 | 109 | 10 | 16 |
| PFOA | C8HF15O2 | 413 | 17.05 | 0.456216 | 1.382472 | y = 0.2022x - 0.0061 | 0.999 | 97 | 85 | 11 | 17 |
| PFNA | C9HF17O2 | 463 | 18.27 | 1.680316 | 5.091868 | y = 0.1088x + 0.0004 | 0.999 | 101 | 102 | 12 | 20 |
| PFDA | C10HF19O2 | 513 | 21.92 | 0.208077 | 0.630537 | y = 0.1674x - 0.005 | 0.972 | 106 | 99 | 11 | 15 |
| PFBS | C4HF9O3S | 299 | 10.90 | 6.925848 | 20.98742 | y = 0.3081x + 0.0037 | 0.999 | 98 | 90 | 9 | 16 |
| PFHxS | C6HF13O3S | 399 | 15.67 | 0.242975 | 0.736289 | y = 0.2344 + 0.0072 | 0.976 | 103 | 114 | 12 | 20 |
| PFOS | C8HF17O3S | 499 | 18.24 | 0.47006 | 1.424425 | y = 0.1144x + 0.0021 | 0.999 | 97 | 101 | 11 | 18 |
| 6-2FTS | C8H5F13O3S | 427 | 16.96 | 0.401824 | 1.217649 | y = 0.0628x + 0.0018 | 0.980 | 99 | 95 | 10 | 15 |
| 8-2FTS | C10H4F17O3S | 527 | 21.15 | 0.433713 | 1.314282 | y = 0.1068x - 5E-05 | 0.999 | 97 | 100 | 12 | 17 |
